# Data-driven design of high-performance MASn_x_Pb_1-x_I_3_ perovskite materials by machine learning and experimental realization

**DOI:** 10.1038/s41377-022-00924-3

**Published:** 2022-07-26

**Authors:** Xia Cai, Fengcai Liu, Anran Yu, Jiajun Qin, Mohammad Hatamvand, Irfan Ahmed, Jiayan Luo, Yiming Zhang, Hao Zhang, Yiqiang Zhan

**Affiliations:** 1 School of Information Science and Technology, Fudan University, Shanghai, 200433 China; 2 College of Information, Mechanical and Electrical Engineering, Shanghai Normal University, Shanghai, 200234 China; 3 Center of Micro‐Nano System, Fudan University, Shanghai, 200433 China; 4 Department of Physics, Chemistry and Biology, Linköping University, Linköping, SE-58183 Sweden; 5 Key Laboratory of Micro and Nano Photonic Structures and Department of Optical Science and Engineering, Fudan University, Shanghai, 200433 China; 6 Yiwu Research Institute of Fudan University, Chengbei Road, Yiwu City, Zhejiang 322000 China

**Keywords:** Solar energy and photovoltaic technology, Electronics, photonics and device physics

## Abstract

The photovoltaic performance of perovskite solar cell is determined by multiple interrelated factors, such as perovskite compositions, electronic properties of each transport layer and fabrication parameters, which makes it rather challenging for optimization of device performances and discovery of underlying mechanisms. Here, we propose and realize a novel machine learning approach based on forward-reverse framework to establish the relationship between key parameters and photovoltaic performance in high-profile MASn_x_Pb_1-x_I_3_ perovskite materials. The proposed method establishes the asymmetrically bowing relationship between band gap and Sn composition, which is precisely verified by our experiments. Based on the analysis of structural evolution and SHAP library, the rapid-change region and low-bandgap plateau region for small and large Sn composition are explained, respectively. By establishing the models for photovoltaic parameters of working photovoltaic devices, the deviation of short-circuit current and open-circuit voltage with band gap in defective-zone and low-bandgap-plateau regions from Shockley-Queisser theory is captured by our models, and the former is due to the deep-level traps formed by crystallographic distortion and the latter is due to the enhanced susceptibility by increased Sn^4+^ content. The more difficulty for hole extraction than electron is also concluded in the models and the prediction curve of power conversion efficiency is in a good agreement with Shockley-Queisser limit. With the help of search and optimization algorithms, an optimized Sn:Pb composition ratio near 0.6 is finally obtained for high-performance perovskite solar cells, then verified by our experiments. Our constructive method could also be applicable to other material optimization and efficient device development.

## Abstract (web summary)

The forward-reverse framework based on machine learning for MASn_x_Pb_1-x_I_3_ perovskite solar cells is reported. The practicability of bandgap model revealing asymmetrically-bowing shape and optimized Sn:Pb ratio are verified by experiments.

## Graphical abstract

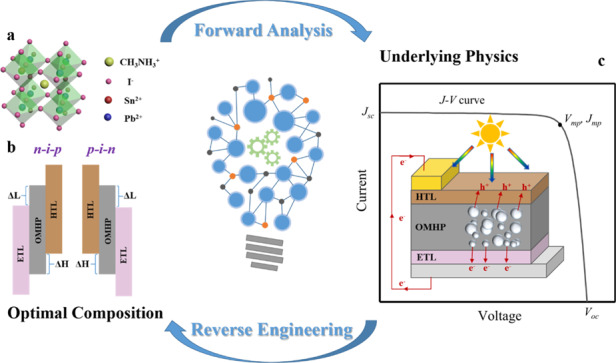

## Introduction

Since the perovskite solar cells (PSCs) are proposed by Kojima et al. in 2009^1^, they have been studied extensively with a rapid rise in power conversion efficiency (PCE)^2–5^ which exceeds 25.5% in single-junction PSC^6^. Organic metal halide perovskites (OMHPs) bestowed with outstanding optoelectronic properties are beneficial for high-performance PSCs, including tunable band gap, low exciton binding energy, high light absorption coefficient and long carrier diffusion length^7–10^. One of the most typical OMHPs is methylammonium lead tri‐halide (MAPbI_3_), which has attracted intensive interest and is one of the most promising materials for developing low-cost and solution-processed optoelectronic technology^4,11^. Despite the rising efficiency records for PSC devices, the efficiency for single-junction MAPbI_3_-based device is limited by the band gap (≈1.6 eV), which is higher than the optimal range of Shockley-Queisser (S-Q) limit (≈1.35 eV)^12^. To exceed the S-Q limit, recently all-perovskite tandem solar cells (PTSCs) composed of wide-bandgap subcells and low-bandgap subcells have been proposed and proven to possibly further increase the efficiency, considering improved utilization of solar energy^13,14^. Tin (Sn), as an environmentally-safer element in the same group of periodic table with Pb, can replace Pb in MAPbI_3_ crystal partially or completely to form Sn-Pb alloying or Sn-based crystals, i.e. MASn_x_Pb_1-x_I_3_^13^, which can tune the band gap of Sn-Pb alloys between 1.1 eV and 1.6 eV by varying stoichiometric ratios of Sn to Pb^15–18^. Therefore, MASn_x_Pb_1-x_I_3_ has been considered as the most promising candidate for high-efficiency single-junction PSCs or low-bandgap subcells in PTSC, responsible for absorbing low-energy photons^13,19–21^. Much effort has been devoted and the efficiency for mixed Sn-Pb PSCs has increased to 18.6% in PTSCs with MA as A site^22^, and 23.3% in Sn-Pb PSC with MA-FA-Cs as A site^23^, which is approaching the recorded efficiency achieved in single-junction Pb-based PSCs. However, for the goal of exceeding the efficiency of Pb-based PSCs and reaching S-Q limit in the future, it still needs a lot of optimization. The unsatisfactory performance for mixed Sn-Pb PSCs is mainly due to the poor morphology of fabricated Sn-based perovskites resulted from Sn vacancies formed by easy oxidation of Sn^2+^ to Sn^4+^ in ambient environment^20,24^, and partially hindered by the insufficient understanding of the photovoltaic properties of mixed Sn-Pb perovskites, such as the underlying mechanisms for manipulations of band gap and photovoltaic-related parameters.

Current investigations of high-efficiency PSC devices require delicate control of chemical synthesis, laborious experimental steps, substantial resource input and a long research cycle to optimize the perovskite compositions, material of each transport layer, interfacial changes and other related parameters, with the purpose to establish the relationship between desirable PSC properties and fabrication parameters. However, due to the huge chemical space for mixed Sn-Pb alloys, the trial-error experiments are tedious as well as time and energy consuming, and sometimes it is beyond reach of providing a thorough investigation. Machine learning (ML) as a new tool learning from known data to solve intractable and complicated problems, can establish complex nonlinear relationship between input parameters and output property to make rapid prediction without prior knowledge^25^. Moreover, with increasing amount of experimental data, the established model could be continuously optimized and its prediction ability could be further improved. At present, there are reports on the prediction of perovskite band gap using ML method^26–30^, and some published studies have used ML to design organic solar cells (OPVs) and predict the performance of OPVs^31–33^. Sahu et al. used 13 important microscopic properties of organic materials to build a ML model for predicting PCE of OPVs and constructed a dataset for 280 small molecule OPV systems^34^. David et al. utilized a database consisting of 1850 entries of OPV characteristics, performance and stability data and employed a sequential minimal optimization regression model as means of determining the most influential factors governing the solar cell stability and PCE^35^. However, few researches extend similar method to PSCs and ML is rarely used to find the relationship of PSC performance with material properties. Although Odabaşı et al. demonstrated the influencing factors of PSC performance using ML according to a large number of published papers^36^, their work is mainly based on statistical analysis and a clear description about the underlying physics needs to be given. Li et al. predicted the band gap of perovskite materials and PCE of PSCs through ML^37^ and the physical law learned by ML was explored. However, currently most work using ML is purely conducting simulations, and researches on PSCs combined simulations and experiments are still lacking.

In the present work, we propose a forward-reverse framework for the first time, to establish the relationship between key parameters and photovoltaic performances and realize the optimization of photovoltaic performances in mixed Sn-Pb PSCs. In the process of forward design, firstly, we establish a ML model that predicts the band gap (*E*_*g*_) of OMHP materials with varied composition, and verify the *E*_*g*_ model by experiments. Then, considering the energy levels of OMHP and carrier transport layers, the models that predict PSC performance (short-circuit current *J*_*sc*_, open-circuit voltage *V*_*oc*_, fill factor FF and PCE) are designed and implemented without prior information. The mechanisms behind different performance models are analyzed in detail and several physical rules are concluded as well. Finally, with the goal of high PCE, three features (Sn-Pb ratio, *E*_*g*_ and the publication time of reported article) are used to build PCE model and directly retrodict the optimal OMHP composition. To the best of our knowledge, this process has not been reported on PSCs before. Based on the predicted optimal proportion for mixed Sn-Pb PSCs, we fabricate the samples and the predicted results are verified.

## Results

### Machine learning models

**Fig. 1:**
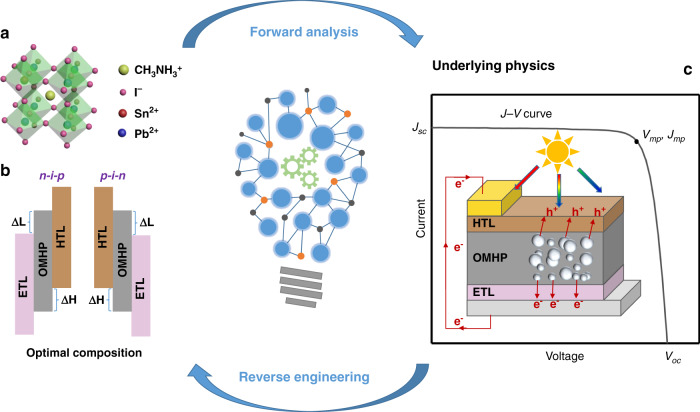
The flow chart of proposed forward–reverse method. For forward analysis. **a** OMHP material information is fed into a machine learning model to predict the band gap; **b** the device structure of regular (n–i–p) and inverted (p–i–n), which represents PSC information and is also combined to construct other ML models. **c** Through ML the performances of PSCs are predicted, and underlying physics could be revealed and analyzed with huge data produced. Then based on the forward results, the optimal composition of OMHP with suitable band gap is reversely deduced for high-performance PSC

The OMHP with formula of ABX_3_ is studied, where the cation A is methylammonium CH_3_NH_3_^+^ (MA^+^), B is the inorganic cation lead (Pb^2+^) or Tin (Sn^2+^), and X is iodide (I^-^), and the basic device structure of PSC can be divided into two types according to the different contact materials with ITO: regular (n–i–p) and inverted (p–i–n)^38^. The device performance is determined by the optical and electrical properties of OMHP, such as exciton binding energy, carrier mobility, the highest occupied molecular orbital (HOMO), the lowest unoccupied molecular orbital (LUMO), etc. The HOMO/LUMO level and interfacial properties of electron transport layer (ETL) and hole transport layer (HTL) could also affect the performance of PSCs. The forward-reverse framework we propose, to study the mixed Sn-Pb perovskites, as shown in Fig. 1, includes two procedures: forward analysis and reverse engineering. The task of forward analysis procedure is to establish the *E*_*g*_ and device-performance models using ML, and that of reverse engineering procedure is to predict the optimization parameters of mixed Sn-Pb perovskites and experimental realization.

We apply five algorithms to different missions in both procedures, including linear regression (LR), support vector regression (SVR), k-nearest neighbor regression (KNR), random forest regression (RFR), gradient boosting regression (GBR) and neural network (NN), which can be implemented by Scikit-learn^39^ and TensorFlow^40^. In the reverse exploration, the established ML model is further inversely analyzed to carry out a search of maximum PCE value under some restrictions using genetic algorithm (GA) or Bayesian optimization algorithm (BO), which are implemented by Python packages (bayesian-optimization^41^ and deap^42^). Related detailed explanations of these algorithms are provided in Supplementary notes.

Special care should be taken when preparing the data in ML modeling. During the preparation of ML dataset for *E*_*g*_ model, the repeated data points with the same Sn-Pb ratio and *E*_*g*_ is counted once, and if a given ratio corresponds to different reported values of *E*_*g*_, we keep all of those values to avoid data bias. For example, MASn_0.75_Pb_0.25_I_3_ has four reported *E*_*g*_ values, i.e. two 1.18 eV, one 1.17 eV and one 1.27 eV. We keep one data point for 1.18 eV and two data points for 1.17 eV and 1.27 eV. To explore which elemental property under different Sn-Pb ratios is more dominant in determining the *E*_*g*_ of MASn_x_Pb_1-x_I_3_, 14 physicochemical parameters^43^ are used as descriptors of inputs for *E*_*g*_ regression model (see Table S1), and the SHAP (Shapley Additive exPlanations) method^44^ is used to obtain the contribution of each feature to the *E*_*g*_ model.

When building the ML model to predict photovoltaic performance of PSCs including *J*_*sc*_, *V*_*oc*_, FF and PCE, three parameters are used as input parameters: i) the band gap (*E*_*g*_) of OMHP material, ii) the energy difference (*ΔH*) between the HOMO of HTL and OMHP material, iii) the energy difference (*ΔL*) between the LUMO of ETL and OMHP material. The schematic energy band diagrams of n–i–p and p–i–n structures are presented in Fig. 1. For n–i–p or p–i–n, *ΔH* and *ΔL* are both calculated by $\Delta {{{\mathrm{H}}}} = {{{\mathrm{HTL}}}}_{{{{\mathrm{HOMO}}}}} - {{{\mathrm{OMHP}}}}_{{{{\mathrm{HOMO}}}}}$ and $\Delta {{{\mathrm{L}}}} = {{{\mathrm{OMHP}}}}_{{{{\mathrm{LUMO}}}}} - {{{\mathrm{ETL}}}}_{{{{\mathrm{LUMO}}}}}$.

Before reverse design, it is important to rebuild the PCE model for PSCs that involves features including Sn-Pb ratio, the *E*_*g*_ of OMHP and annual improvement of material qualities and optimization of processing (denoted by publication date of research article) inspired from time series prediction. Since there is relatively uniform standard for PCE testing of PSCs, it is meaningful to use publication date as a feature to help the model explore the PCE progress trend of PSCs with passage of time. Due to the difference of preparation technologies, the performance of devices obtained by different research groups at the same ratio could also be different. So we use the maximum PCE result corresponding to each ratio in each year. Then, based on the mentioned model, the virtual design of OMHP materials is carried out by GA or BO.

It should be noted that, the data points are collected from published articles^21,37^ which are randomly divided into a training set and a test set in ratios of 90% and 10%. The dataset for *ΔH* and *ΔL* used to build the PCE model is shown in Figure S1 with 181 data points. To build the *E*_*g*_ prediction model, 43 data points are used from the performance model after discarding duplicate material composition data with the same reported value of *E*_*g*_. In the model of reverse exploration, the data points with best PCE for a given preparation method in different publication times are chosen. For these models, we use 5-fold cross validation to optimize the hyper parameters of ML algorithm based on a Python library called hyperopt^45^, which could effectively speed up the search of the values of multiple hyper parameters. The test subset is only used to test the quality of the built model. We evaluate the performance of constructed models by three indicators (the coefficient of determination R^2^, root mean square error RMSE and mean absolute error MAE).

### Material design by ML: band gap

**Fig. 2:**
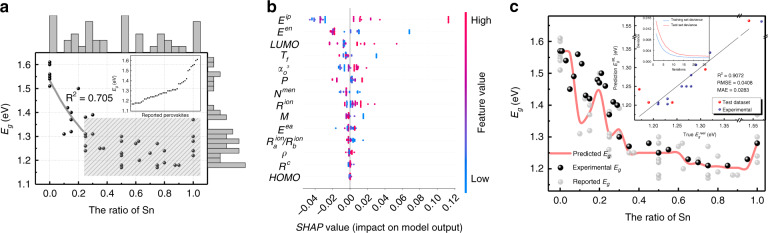
The results for band gap (*E*_*g*_) model. **a** The marginal histograms and plot of *E*_*g*_ versus Sn-Pb ratio. The black dots are collected reported data points, and the gray line is the result of traditional fitting. The bad fitting index (0.705) and the disorder shaded part indicates that it is difficult to find the relationship between Sn-Pb ratio and *E*_*g*_ by traditional method. The inset is band gap distribution of 43 collected perovskites. **b** The feature importance ranking produced from GBR and SHAP library with 14 inputs, showing the elemental properties in descending order of importance (rank). The x-axis labeled as the SHAP value represents the impact on *E*_*g*_ value. The red and blue color indicate high and low values of a given feature, respectively. The top five features which are most important on the formation of *E*_*g*_ are weighted first ionization energy *E*^*ip*^, Mulliken’s electronegativity of B-site *E*^*en*^, LUMO, tolerance factor *T*_*f*_ and unit cell lattice edge $\alpha _o^3$, respectively. **c** The comparison of the predicted *E*_*g*_ values using Sn-Pb ratio as input and the experimentally measured values. The light gray dots are collected reported data points for comparison. The inset shows actual values versus predicted results by GBR model for test set and our experimental samples marked with red and blue dots, respectively. The black line represents the ideal situation of the prediction (predicted results are equal to actual values). The smaller the distance between data point and black line, the better and more reliable the prediction. The subplot of inset shows the convergence of model accuracy

To utilize the solar energy to the maximum extent, the minimum *E*_*g*_ of mixed Sn-Pb perovskites used as the low-bandgap subcells to absorb low-energy photons, are critical to maximize the PCE of PTSCs. Moreover, manipulating the *E*_*g*_ by an appropriate ratio is also critical for the realization of high-efficiency and environmentally friendly single-junction PSCs based on mixed Sn-Pb perovskites. As previously reported, the valence bands (VBs) of MAPbI_3_/MASnI_3_ crystals are formed by the antibonding states of I-*p* and Pb/Sn-*s* atomic orbitals, and the conduction band (CBs) are formed by the antibonding states of I-*p* and Pb/Sn-*p* atomic orbitals. When the ratio of Sn increases in MASn_x_Pb_1-x_I_3_ alloys, the VB/CB levels undergo the shift from −5.45 eV/-3.90 eV (x = 0) to −5.47 eV/-4.17 eV (x = 1) respectively^16^ and Pb-*p* as CB and antibonding states of I-*p* and Sn-*p* as VB, which subsequently leads to the well-known bowing effect in the *E*_*g*_ of MASn_x_Pb_1-x_I_3_. However, the previously reported *E*_*g*_ values denoted as black dots in Fig. 2a are not directly linearly or parabolically dependent on the ratio x, since replacing of Pb by Sn will cause lattice compression and octahedral tilting in MASn_x_Pb_1-x_I_3_ structure, and increasing the former or enhancing the latter will increase or decrease the *E*_*g*_^13^. To reveal the underlying mechanism of the *E*_*g*_ in mixed Sn-Pb perovskites, we retrieve the contributions from elemental properties and structural information of mixed Sn-Pb perovskites by building the ML-bandgap model using 14 physicochemical parameters listed in Table S1 as inputs.

Due to the limited available *E*_*g*_ data, we only use traditional ML method without NN. Five ML methods (LR, SVR, KNR, RFR and GBR) are used to build the *E*_*g*_ model with 14 features as input and the results are listed in Table 1. The GBR algorithm performs best with the corresponding values of R^2^, RMSE and MAE of 0.9172, 0.0386, and 0.0325, respectively. The SHAP method is used to further interpret the GBR-bandgap model^44^. The calculated feature importance ranking produced from the GBR and SHAP library is shown in Fig. 2b, with x-axis labeled as the SHAP value representing the impact on *E*_*g*_ value, and the red and blue colors indicating high and low values of a given feature, respectively. The top five features which are most important on the formation of *E*_*g*_ are weighted first ionization energy *E*^*ip*^, Mulliken’s electronegativity of B-site *E*^*en*^, LUMO, tolerance factor *T*_*f*_ and unit cell lattice edge $\alpha _o^3$, respectively. Obviously, LUMO plays a more important role than HOMO does, which is in good agreement with the large energy difference of -0.27 eV in CB and the small one of -0.02 eV in VB when Pb is replaced by Sn in MASn_x_Pb_1-x_I_3_ alloys mentioned above. In fact, when replacing Pb by Sn, *E*^*en*^ and *T*_*f*_ increase, and *E*^*ip*^, LUMO and $\alpha _o^3$ decrease. However, as shown in Fig. 2b, only the decreasing *E*^*ip*^ and increasing *E*^*en*^ clearly decrease the *E*_*g*_. The decreasing LUMO when the ratio of Sn increases leads to the simultaneous increase and decrease of *E*_*g*_, which is consistent with the bowing effects as shown in Fig. 2a. In a similar way, the replacing of big Pb atoms by small Sn atoms changes the structural characteristics, and subsequently decreases $\alpha _o^3$ and increases *T*_*f*_, which also leads to the simultaneous increase and decrease of *E*_*g*_, as shown in Fig. 2b.

**Table 1:** The comparison of prediction performance of *E*_*g*_ models with different features and ML regression algorithms by three evaluation metrics (R^2^, RMSE and MAE). The best results are highlighted in bold.

Method	14 features			One feature		
	R^2^	RMSE	MAE	R^2^	RMSE	MAE
LR	0.6864	0.0751	0.0630	0.6064	0.0841	0.0707
SVR	0.8775	0.0469	0.0422	0.8221	0.0565	0.0516
KNR	0.8997	0.0425	0.0340	0.8833	0.0458	0.0365
RFR	0.9105	0.0401	**0.0284**	0.9065	0.0410	0.0344
GBR	**0.9172**	**0.0386**	0.0325	**0.9072**	**0.0408**	**0.0283**

To reduce the complexity and facilitate the PSC-device design, we further build a new *E*_*g*_ model based on one-dimensional feature (Sn-Pb ratio) as input. In this case, the GBR algorithm also performs best with the corresponding values of R^2^, RMSE and MAE of 0.9072, 0.0408, and 0.0283, respectively, as listed in Table 1. To verify the precision of our new ML-bandgap model, we experimentally fabricate a series of new mixed Sn-Pb perovskite samples with various compositions. The measured and GBR-predicted *E*_*g*_ along with reported results is shown as inset in Fig. 2c, with blue dots denoting our experimental results and red dots denoting previously reported results. The experimental *E*_*g*_ values are deduced by Tauc plot (Fig. S2). It shows that our trained *E*_*g*_ model could not only predict the data in test set, but also accurately predict *E*_*g*_ of the new MASn_x_Pb_1-x_I_3_ samples. As previously mentioned, the replacing of Pb by Sn leads to significant changes in structure, e.g. lattice compression, octahedral tilting, and in electronic properties, e.g. VB/CB reconstructing, which thus results in a complicated dependence of *E*_*g*_ on Sn ratio in mixed Sn-Pb perovskites. To further reveal the dependence of *E*_*g*_ on Sn ratio, as shown in Fig. 2c, the ratio-dependent *E*_*g*_ for mixed Sn-Pb perovskite predicted by our new ML-bandgap model (red line), which match well with our fabricated ones (black dots), manifests itself with asymmetrically bowing shape and a minimum value of 1.198 eV of at the Sn ratio of 93.3%. The optimized Sn ratio for the S-Q limit at the *E*_*g*_ of 1.35 eV is predicted to be 10.0%, 12.2% and 23.3%.

### Perovskite solar cell design: photovoltaic performance

As mentioned above, both n-i-p and p-i-n PSC devices are under study here as shown in Fig. 1b and are composed of a thin-layer photoactive OMHP absorbers sandwiched between two transport layers ETL and HTL, which are used to selectively transport electrons and holes to cathode and anode respectively. Ideally, incident photons are absorbed with nearly unity efficiency by OMHP film, and densities of photo-generated nonequilibrium free carriers in CB and VB can be evaluated by the quasi-Fermi level splitting (QFLS), i.e. $QFLS = E_F^e - E_F^h$ where $E_F^{e/h}$ is the quasi-Fermi level for electrons and holes respectively, which is also the open-circuit voltage (*V*_*oc*_) of the PSC devices in the S-Q theory. The values of *V*_*oc*_ are deterioted mainly by the unwanted nonradiative combinations occurring at the surface defects or grain boundaries in the OMHP film. Then the photo-generated electrons and holes are selectively extracted by the ETL and HTL to the cathode and anode respectively, and generate currents, e.g. *J*_*sc*_ at short-circuit condition. The extraction efficiency of carriers from OMHP film is mainly affected by the surface qualities and the energy differences between OMHP and HTL/ETL, i.e. *ΔH* (*ΔL*). The values of *ΔH* (*ΔL*) are generally larger than or equal to zero, since negative ones will introduce extraction barriers between OMHP film and transport layers, but they can not be too large to induce significant energy loss at the interface and form the transport barriers between transport layers and electrodes, which in turn may decrease *J*_*sc*_. For example, in our case, the HOMO and LUMO of OMHP are -5.4 eV and -3.9 eV respectively, and the work functions of working anode (Au)/cathode (Al) are -5.1 eV and -4.3 eV, respectively. When *ΔH* (*ΔL*) between HTL (ETL) and OMHP film is higher than 0.3 (0.4) eV, HTL-anode (ETL-cathode) extraction potential barriers is generated. Generally, the open-circuit voltage *V*_*oc*_ relates to the short-circuit current *J*_*sc*_ by the expression,

1 $$V_{oc} = \frac{{n_{id}k_BT}}{q}\ln \left( {\frac{{J_{sc}}}{{j_0}} + 1} \right)$$

where *n*_*id*_ is the ideality factor and *j*_*0*_ is the dark generation current. The electric power is zero at both open- and short-circuit conditions, and reaches a maximum power point at which the voltage *V*_*mp*_ and current *J*_*mp*_ give the fill factor FF of a PSC device, i.e. FF = *V*_*mp*_*J*_*mp*_/*J*_*sc*_*V*_*oc*_. Empirically FF can be written as^46^,

2 $${{{\mathrm{FF}}}} = \frac{{v_m}}{{v_m + 1}}\frac{{v_{oc} - \ln \left( {v_m + 1} \right)}}{{v_{oc}\left( {1 - e^{ - v_{oc}}} \right)}}$$

where $v_{ov} = V_{oc}/n_{id}k_BT$ and $v_m = v_{oc} - \ln \left( {v_{oc} + 1 - \ln v_{oc}} \right)$. Subsequently PCE is given by $\eta _{PCE} = J_{sc} \times {{{\mathrm{FF}}}} \times V_{oc}/P_{sun}$, where *P*_*sun*_ is the total incoming solar energy. For the mixed Sn-Pb perovskite as the OMHP film in PSC under study here, the increasing of Sn introduces more Sn vacancies and oxided Sn^4+^ in the OHMP, which may enhance the non-radiative recombination via Shockley-Read-Hall and Auger recombination processes at the defects, decreasing *V*_*oc*_ and *J*_*sc*_, even though *E*_*g*_ is tuned to the optimized value predicted by the S-Q theory. Therefore, to achieve the maixmum PCE for MASn_x_Pb_1-x_I_3_-based PSC devices, a detailed optimization process regarding the influence on *J*_*sc*_, *V*_*oc*_ and PCE from *E*_*g*_, *ΔH* and *ΔL* is necessary.

Similiar to the aforementioned process, the influences of *E*_*g*_, *ΔH* and *ΔL* on photovoltaic performances of mixed Sn-Pb PSC devices, i.e. *V*_*oc*_, *J*_*sc*_, FF and PCE, are investigated using ML methods, and the performances of different ML algorithms are listed in Table 2, in which experimental *E*_*g*_, *ΔH* and *ΔL* are used as inputs, as comparison to those using predicted *E*_*g*_ as inputs listed in Table S2. In both simulations, NN behaves better than others, which confirms the superiority of NN in dealing with complex cases. By comparison, we find that, for *J*_*sc*_, using the predicted *E*_*g*_ as feature, the model metrics is improved, but for FF model, using the predicted *E*_*g*_ will worsen the model. For PCE and *V*_*oc*_ models, whether the predicted *E*_*g*_ is used, the model results change little. In addition, no matter what kind of situation and target, the values of R^2^ of all models are significantly greater than 0.5, which means the built models can give relatively accurate prediction. The reason for large variation of metrics in FF with different models might be that the factors affecting FF are complex, and the dependence on involved features is beyond our collected data. The performances of NN in our dataset for four targets are shown in Figure S3, and the descent process of training/test set loss values during NN training is provided in Figure S4, both of which verify the convergence of our NN model. Herein, we set *ΔH* and *ΔL* from −0.35 eV to 1.00 eV and *E*_*g*_ from 1.15 eV to 1.65 eV in the prediction set.

**Table 2:** The prediction performance of different regression algorithms for four targets (FF, *J*_*sc*_, *V*_*oc*_ and PCE) in designing PSCs device using experimental *E*_*g*_, *ΔH* and *ΔL* as inputs

Method	FF			*J*_*sc*_			*V*_*oc*_			PCE		
	R^2^	RMSE	MAE	R^2^	RMSE	MAE	R^2^	RMSE	MAE	R^2^	RMSE	MAE
LR	0.4750	14.3140	8.1743	−0.0676	5.3079	2.9698	0.6047	0.1345	0.0968	0.3422	4.0129	2.8447
SVR	0.2937	16.6026	7.4239	0.1519	4.7310	2.4441	0.4389	0.1602	0.1084	0.5518	3.3124	2.2708
KNR	0.4580	14.5433	6.9755	0.7913	2.3471	1.8209	0.8022	0.0951	0.0557	0.6229	3.0385	2.5294
RFR	0.5698	12.9566	6.9801	0.5262	3.5362	2.3123	0.6068	0.1341	0.0936	0.6598	2.8858	2.2669
GBR	0.5477	13.2863	7.1833	0.0239	5.0754	2.7716	0.6873	0.1196	0.0884	0.7651	2.3982	1.8816
NN	**0.9096**	**4.5641**	**3.4786**	**0.8378**	**2.6438**	**1.6301**	**0.9024**	**0.0347**	**0.0289**	**0.9026**	**1.9626**	**1.5439**

The best results are highlighted in bold and NN behaves the best among competitive algorithms due to higher R^2^ and smaller RMSE and MAE

**Fig. 3:**
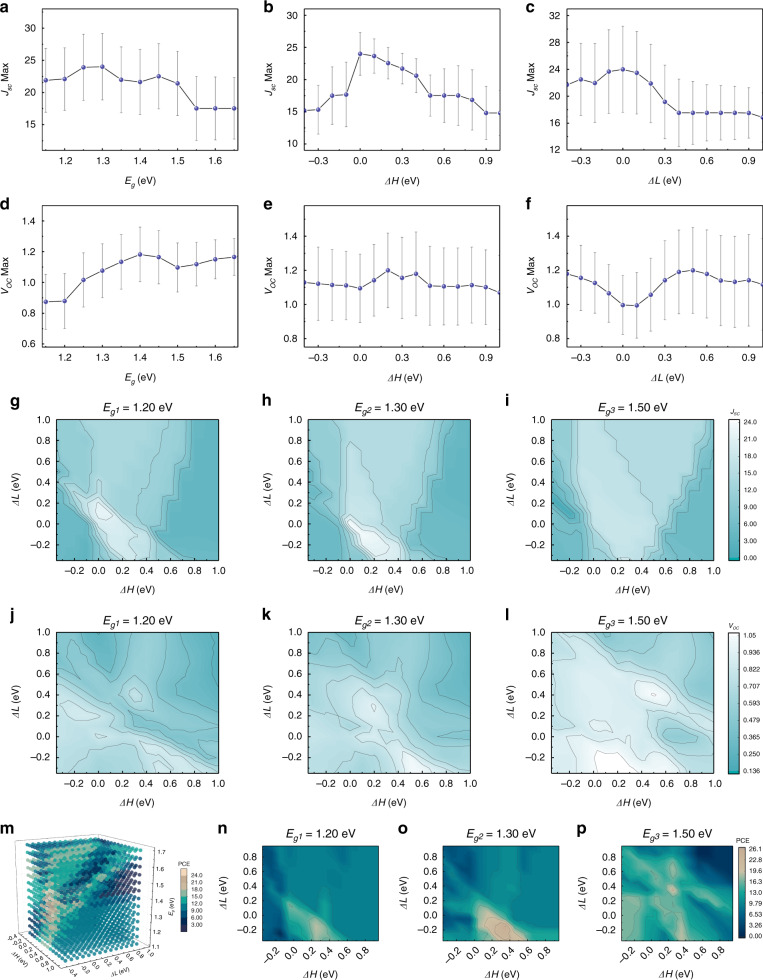
Further analysis of PSCs performance behind NN model with three parameters as input (the *E*_*g*_ of OMHP material, the energy difference *ΔH* and *ΔL*). **a** The maximum *J*_*sc*_ versus *E*_*g*_; **b** the maximum *J*_*sc*_ versus *ΔH*; **c** the maximum *J*_*sc*_ versus *ΔL*; **d** the maximum *V*_*oc*_ versus *E*_*g*_; **e** the maximum *V*_*oc*_ versus *ΔH*; **f** the maximum *V*_*oc*_ versus *ΔL*. 2D-contour map of *J*_*sc*_ predictions at **g** *E*_*g1*_; **h** *E*_*g2*_; **i** *E*_*g3*_, and 2D-contour map of *V*_*oc*_ predictions at **j** *E*_*g1*_; **k** *E*_*g2*_; **l** *E*_*g3*_. **m** 4D-scatter plot of PCE with respect to *E*_*g*_, *ΔH*, and *ΔL* exhibiting that the highest PCE value is in the range of 1.30 eV to 1.40 eV. 2D-contour map of PCE predictions at **n** *E*_*g1*_; **o** *E*_*g2*_; **p** *E*_*g3*_. Here, *E*_*g1*_ = 1.20 eV, *E*_*g2*_ = 1.30 eV, and *E*_*g3*_ = 1.50 eV

The relationships of *J*_*sc*_ and *V*_*oc*_ with *E*_*g*_ are shown in Fig. 3, with the bar indicating standard deviation. As we know, in the S-Q theory, *J*_*sc*_ deceases and *V*_*oc*_ increases when *E*_*g*_ increase, where nonradiative recombination are totally neglected. However, as shown in Fig. 3a, in the *E*_*g*_ regions ranging roughly from 1.15 eV to around 1.25 eV and 1.40 eV to 1.50 eV, which are corresponding to low-bandgap-plateau (LBP) (rich-Tin, >50% Sn) and defective-zone (DZ) (poor-Tin, <20% Sn) regions respectively, *J*_*sc*_ increases when *E*_*g*_ increase, which are consistent with the unchanged or decreasing *V*_*oc*_ with increasing *E*_*g*_ as shown in Fig. 3d. The deviation observed in predicted *J*_*sc*_ and *V*_*oc*_ from the S-Q theory captured in our model, which was experimentally observed recently in ((HC(NH_2_)_2_)_0.83_Cs_0.17_)(Pb_1-y_Sn_y_)I_3_ family of perovskite materials as well^47^, is due to the significant nonradiative recombination at the deep level traps induced by structural disorders (DZ) and enhanced susceptibility of Sn^2+^ to oxidation (LBP). Similar to the situation in ((HC(NH_2_)_2_)_0.83_Cs_0.17_)(Pb_1-y_Sn_y_)I_3_ perovskites, in the poor-Tin (DZ) region, the replacing of Sn in neat Pb perovskites induces local heterogeneity around Sn sites along with overall lattice compression and octahedral tilting, and such crystallographic distortion leads to energetic disorder near the band edges accommodating deeper traps formation, accompanied with a rapid change in *E*_*g*_ as shown in Fig. 2a. Therefore, the nonradiative *J*_*sc*_ are enhanced in the DZ region. However, in the rich-Tin (LBP) region, due to the cease of volume compression in the compositional perovskites, crystallographic and electronic stabilities are achieved, as shown in Fig. 2a as well. Since Sn content is beyond 50% in the LBP region, the subsequently enhanced electric susceptibility resulted from increased Sn^4+^ content also deteriorates the photovoltaic performances (*J*_*sc*_ and *V*_*oc*_).

For *ΔH* and *ΔL*, the photo-generated carrier transport is not only affected by extraction barriers *Δ*, but also by the type of transport layer material, fabrication processing, etc. Thus it is complicated to obtain optimized *J*_*sc*_ and *V*_*oc*_ by manipulating *ΔH* and *ΔL*. As shown in Fig. 3b, c, the changes of *J*_*sc*_ with *ΔH* and *ΔL* are similar and it is not conducive to *J*_*sc*_ as *ΔH* (*ΔL*) is too large or too small. However, as shown in Fig. 3e, f, different from *J*_*sc*_, the maximum value of *V*_*oc*_ does not appear in the region where *ΔH* (*ΔL*) is equal to zero. To further analyze the extraction barrier related to *J*_*sc*_ and *V*_*oc*_ with different fixed *E*_*g*_ values in detail, 2D-contour maps of maximum *J*_*sc*_ and *V*_*oc*_ prediction with different *E*_*g*_ are calculated and shown in Fig. 3g–l, where x-axis and y-axis represent *ΔH* and *ΔL*, respectively. The lighter color indicates the higher *J*_*sc*_ and *V*_*oc*_. And for simplication, the values of *E*_*g*_ are chosen as 1.20 eV, 1.30 eV and 1.50 eV to conduct further analysis, which belong to the LBP, normal and DZ regions, respectively.

For *J*_*sc*_ as shown in Fig. 3g–i, as the *E*_*g*_ increase from 1.20 eV→1.30 eV→1.50 eV, the values of *J*_*sc*_ overally decreases, which is consistent with the S-Q theory. When *ΔH* (*ΔL*) is much lower than zero, it means there is a big potential barrier between OMHP material and HTL (ETL), which will block the direct transfer of carriers and consequently decrease the current. When *ΔH* (*ΔL*) is much higher than zero, it could increase the speed of the carriers as they pass through transport layer, but this could also lead to induce energy loss at the interface and transport barrier between the transport layer and the electrode, and thus decreasing *J*_*sc*_. Figure 3g–i also show that, when *ΔL* is negative, it is possible to manipulate *ΔH* to achieve the maximum *J*_*sc*_, but not vice versa. Take Fig. 3h as an example. The maximum *J*_*sc*_ appears at *ΔH* = 0.2 eV and *ΔL* = -0.2 eV, i.e, there is 0.2 eV barrier between ETL and OMHP and 0.2 eV positive energy difference between HTL and OMHP. In this condition, the maximum *J*_*sc*_ of 21.7 mA/cm^2^ achieves. Conversely, for *ΔH* = -0.2 eV and *ΔL* = 0.2 eV eV, the value of *J*_*sc*_ drops (2.5 times) to 9.8 mA/cm^2^, which should be due to the more difficult extraction for holes in HTL compared with electron extraction in ETL, since holes possess larger effective mass and smaller mobility compared to electrons^48^. Therefore in the actual PSC design, employing excellent hole transport would lead to high photovoltaic performance^49,50^.

For *V*_*oc*_ as shown in Fig. 3j–l, as the *E*_*g*_ increase from 1.20 eV→1.30 eV→1.50 eV, the values of *V*_*oc*_ overally increase, which is consistent with the S-Q theory as well. The difficulty of hole extraction in HTL can also be observed. For example, as shown in Fig. 3l, the maximum *V*_*oc*_ appears in the region of *ΔH* = 0.2 eV and *ΔL* = -0.2 eV, which means 0.2 eV barrier between ETL and OMHP can promote the accumulation of electrons at the interface subsequently enlarging QFLS within OMHP and thus *V*_*oc*_. Because the transport of holes in OMHP is more difficult than electrons, positive *ΔH* is required to facilitate the hole extraction, which could reduce the concentration of holes in OMHP thus reducing unwanted nonradiative recombination.

**Fig. 4:**
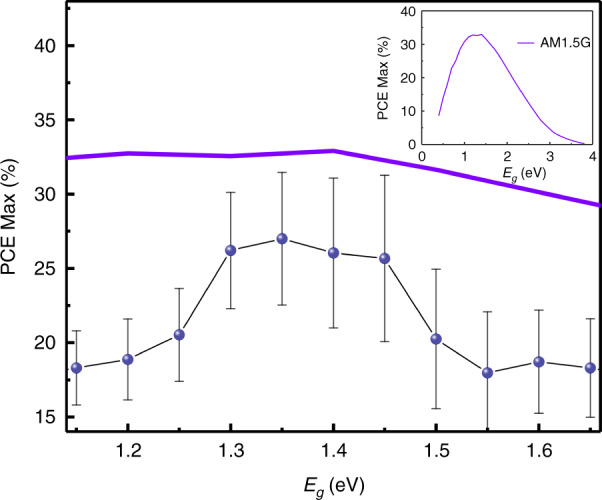
The obtained relationship between PCE and *E*_*g*_ from different ways. The black and violet lines represent the maximumpredicted PCE with regard to *E*_*g*_ and the theoretical limit of PCE fromShockley–Queisser (S–Q) limit, respectively. The inset is the reduction curveof S–Q limit.

For the goal PCE, Fig. 3m shows a 4D-scatter plot of predicted PCE based on NN algorithm, where *ΔH* and *ΔL* are changed from -0.35 eV to 1.00 eV and *E*_*g*_ is changed from 1.15 eV to 1.65 eV, which reveals that, the highest PCE values are in the range of 1.30 eV to 1.40 eV. To further analyze the relationship between PCE and *E*_*g*_, the maximum PCE of each *E*_*g*_ is extracted from Fig. 3m and plotted in Fig. 4, where the theoretical prediction by S-Q theory under AM1.5 G radiation is also shown. Both S-Q limit and our ML results show that the ideal *E*_*g*_ of 1.35 eV^12,51,52^, and the trend of both lines is also consistent, which is an astonishing finding since only data from MASn_x_Pb_1-x_I_3_ system are used in our model without providing any information about the solar spectrum. The peak at about 1.60 eV is probably due to the extensive use of this material by researchers and the continuous optimization achieved for the performance of PSCs on this material.

In addition, in order to obtain the relationship of PCE with *ΔH* and *ΔL* in the different values of *E*_*g*_, 2D-contour maps are also drawn in Fig. 3n–p. The region of highest PCE shifting from smaller *ΔH*, *ΔL* to higher *ΔH*, *ΔL* with the increase of *E*_*g*_ of OMHP material, was also observed in perovskite materials of ABX_3_-type (A=MA/FA/Cs^+^, B=Pb/Sn^2+^, X=Br/Cl/I^-^)^37^, which manifests the extensibility of our ML model to general OMHP systems. Furthermore, it is interesting that as we compare Fig. 3n–p with Fig. 3g–l, the improvement of goal PCE at 1.30 eV (near optimal *E*_*g*_) is mainly due to enhanced *J*_*sc*_ because both maximum region and contour overlap. For comparison to further check our model, according to Eq. (2), the filling factor FF can be obtained with ML-predicted *V*_*oc*_ values and the ideality factor *n*_*id*_ of 2.5 is deduced according to Eq. (1), and finally PCE can be obtained. The calculated dependence of PCE on *E*_*g*_, *ΔH* and *ΔL* is shown as Figure S5, which is reasonably consistent with the ML-predicted results as shown in Fig. 3n–p.

### Reverse engineering

In order to further verify the effectiveness of ML, we have performed experimental tests. Through forward analysis, the process of OMHP material selection and PSC design are completed, but the relationship between Sn-Pb ratio and PCE of PSC cannot be directly given, that is, to determine which ratio could give the corresponding maximum predicted PCE. Therefore, in this section, a data-driven reverse engineering is proposed to explore potential property of MASn_x_Pb_1-x_I_3_ material used in PSCs based on one-step spin coating process and inverted structure.

Due to the reduction of dataset resulting from the limit of spin coating process and device structure, as previously mentioned, three features of Sn-Pb ratio, *E*_*g*_ and the publication time of reported article are taken to build a new PCE model based on GBR algorithm. Then the established GBR model is further inversely analyzed to lead a search of maximum PCE using BO and GA. The search conditions of 10 initial points alongwith 20 iterations, and the population size of 100 alongwith 20 generations are used for BO and GA, respectively. The element Sn in the B-site has the doping ratio from 0.5 to 1.0 with step 0.001, and the second element Pb is given the remaining doping ratio, because the *E*_*g*_ obtained by doping Sn in this range has the opportunity to obtain the maximum PCE according to previous forward analysis, while reducing the content of Pb as much as possible to reduce the toxicity of obtained OMHP. Figure S6 shows the fitness of PCE to the search round and shows how it changes with the optimization variable. GA presents a more stable and efficient exploration process than BO. However, both GA and BO give very close maximum PCE, i.e., 18.23% for MA_1.0_Sn_0.624_Pb_0.376_I_3_ by GA and 18.22% for MA_1.0_Sn_0.636_Pb_0.364_I_3_ by BO, respectively.

**Fig. 5:**
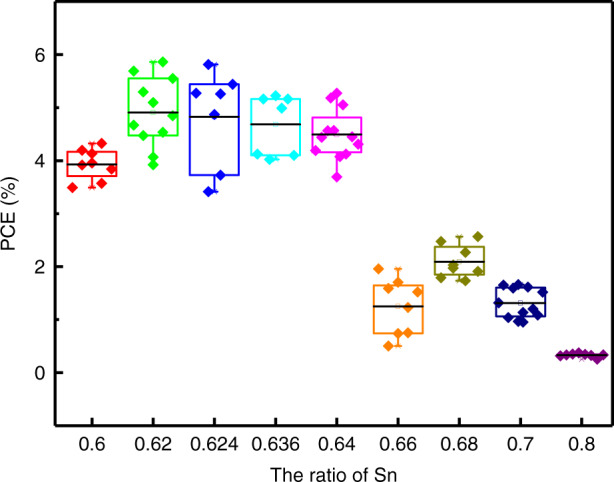
The experimental PCE of MASn_x_Pb_1-x_I_3_ perovskite solar cells with different Sn contents

With the previous settings, we select a series of ratios from 0.5 to 1.0 for experimental validation under the set fabrication processing. As reported in previous works, the perovskite crystallization processes^53^, grain boundary management^54^, interface engineering^55^ and charge transport layer selection^56^ were proved to be critical aspects toward high-efficiency perovskite solar cells. Every precise tuning of above sections in each experimental condition is definitely difficult and time consuming. For now, we do not conduct special optimization for devices and choose a trade-off fabrication process to adapt all conditions to keep the consistency of the processing condition for different ratios to make the horizontal comparability more obvious. Actually, we focus on the curve trend and the obtained results are shown in Fig. 5, which reveals good consistency between experimental data and prediction trend in PCE distribution. The ratios corresponding to the best PCE obtained by GA and BO are near the region of highest experimental result, which illustrates the rationality and accuracy of our model. Moreover, in the range of experimental preparation, the PCE of device deviated from the optimal composition becomes very poor. The corresponding plots of *J*_*sc*_, *V*_*oc*_ and FF for these devices are provided in Figure S7. Compared with the prediction of 18%, the efficiency corresponding to optimal ratio in our experiment still has much room for development that needs further experimental optimization. At present, the best efficiency obtained by Sadhanala et al. in set range of Sn content is 10% at 0.6 that ratio is close to our prediction. Specific data are provided in Table S3. With the accumulation of samples in the future, the optimization measures, such as considering additives, selecting better transport layer materials, etc., should be considered. In addition, our proposed ML approach in the forward-reverse framework can be applied to other perovskites materials with similar structures, such as ABX_3_-type perovskite materials (A = MA/FA/Cs^+^, B = Pb/Sn^2+^, X = Br/Cl/I^-^).

## Discussion

Combining with ML technology, we have proposed an efficient forward-inverse method to research MASn_x_Pb_1-x_I_3_ material and explore high-performance PSCs. We use real experimental data aiming at getting more practical results. During forward analysis, the *E*_*g*_ model of MASn_x_Pb_1-x_I_3_ is first built with 14 physicochemical parameters and Sn-Pb ratio as input respectively, and the asymmetrically bowing relationship between Sn-Pb ratio and *E*_*g*_ of OMHP is found, which match well with our fabricated ones. For the performance models of PSC, the established NN-based models exhibit satisfactory prediction for underlying data points and provide some guidance and new discoveries for PSC devices. The relationship of *J*_*sc*_, *V*_*oc*_ and PCE with *E*_*g*_ of OMHP and energy level difference (*ΔH*/*ΔL*) are analyzed in detail respectively. The relationship of *J*_*sc*_ and *V*_*oc*_ with *E*_*g*_ matches the theoretical results well in most case. The deviation of *J*_*sc*_ and *V*_*oc*_ in LBZ and DZ region from the S-Q theory is also captured by our model, which is experimentally observed recently. The conclusion that hole extraction is more difficult is both obtained in *J*_*sc*_ and *V*_*oc*_ models. And the highest PCE of single-junction PSCs at about 1.35 eV is predicted without any prior information, which is quite consistent with the theoretical result of S-Q limit. Then in the reverse engineering, the optimal ratio of Sn in MASn_x_Pb_1-x_I_3_ within a set range is directly obtained for high-performance PSCs and inference results fit well with experimental validation.

With the proposed target-driven approach, the physical laws obtained are reasonable and the predictions are verified experimentally in MASn_x_Pb_1-x_I_3_ system. Our designed method could be expected to provide deeper understanding of physical phenomena as well as explore new functional materials and high-performance devices. For complex systems involving multiple variables, this method could give results significantly faster. With the accumulation of a database, it could also constantly learn with itself to obtain stronger predictive ability to give more helpful and accurate guidance.

## Materials and methods

### Materials

SnI_2_ (99.999% purity) was purchased from Alfa Aesar. N, N-dimethylform-amide (DMF), dimethyl sulfoxide (DMSO) and SnF_2_ (99% purity) were purchased from Sigma-Aldrich. Methylammonium iodide (MAI) was purchased from Dyesol. Chlorobenzene was purchased from Thermo Fisher. PEDOT:PSS aqueous solution (Al 4083) was purchased from Heraeus Clevios. PbI_2_ was purchased from TCL.

### Mixed tin-lead perovskite film fabrication

A stock solution of 1.4 M MAPbI_3_ solution (i.e. 0% Sn) was prepared by dissolving 1113 mg MAI and 3227 mg PbI_2_ in 5 mL of a mixed solvent of 7:3 DMF:DMSO by volume. A stock solution of 1.4 M MASnI_3_ (i.e. 100% Sn) with 10% molar excess of SnF_2_ with respect to the SnI_2_ content was prepared by dissolving 1113 mg MAI, 2604 mg SnI_2_, 219 mg SnF_2_ in 5 mL of a mixed solvent of 7:3 DMF:DMSO by volume. Solutions with Sn content = 0, 15, 25, 30, 35, 45, 50, 55, 65, 70, 75, 85, 95, 100% were prepared by mixing the 0% and 100% Sn stock solutions in the appropriate ratio. Solutions with Sn content = 62, 62.4, 63.6, 64, 66, 68% were prepared through mixture of the 60% Sn solution with the 70% Sn solution. The precursor solution was stirred for at least 2 h at room temperature. Prior to spin-coating, the perovskite solution was filtered with a 0.22 μm PTFE syringe filter. The perovskite precursors with different Sn content were spin-coated onto the ITO/PEDOT:PSS substrates at 5000 rpm for 30 s. During the spin-coating, 750 μL of toluene was dropped onto the spinning substrates. Then perovskite films were annealed at 100 °C for 10 min to form mixed tin-lead perovskite films.

### Mixed tin-lead perovskite solar cell fabrication

ITO-glass was cleaned using acetone, surfactant, deionized (DI) water, ethanol with ultrasonication for 30 min sequentially and dried with N_2_ flow. ITO-glass was further cleaned by O_2_-plasma treatment for 15 min. PEDOT:PSS aqueous solution was then deposited on the ITO at 4000 rpm for 50 s followed by annealing on a hotplate at 175 °C for 60 min in ambient air. The perovskite films were prepared on the ITO/PEDOT:PSS using mixed tin-lead perovskite film fabrication method described above. After the deposition of the perovskite film, C60 (20 nm)/BCP (3 nm)/Ag (100 nm) were sequentially deposited by thermal evaporation.

### Material characterization

The transmission spectra and absorption spectra were both measured using F20-UV thin-film analyzer (FILMETRICS) at wavelength range between 300 nm and 1100 nm. Tauc plots of absorption spectra to determine the impact of different Sn contents on perovskite band gaps was calculated by absorption spectra.

### Device characterization

The J–V curves of PSCs were measured using a Keithley 2602B source in N_2_ filled glove box at room temperature under AM 1.5 G condition at an intensity of 100 mW/cm^2^, calibrated by a standard Si solar cell (PVM937, Newport). The light source was a 450 watt xenon lamp (Oriel solar simulator, 94023 A). The active area of PSCs was 0.107 cm^2^, defined by the cross of patterned Ag and ITO electrode, and further calibrated by the microscope. The J–V curves were tested both at forward scan (from −0.2 V to 0.7 V, step 0.04 V) without any pre-conditioning before the test.

## Supplementary information

Supplementary Information
