# Supplementary material for: Data-driven design of high-performance MASnxPb1-xI3 perovskite materials by machine learning and experimental realization
Source: Light Sci Appl. 2022 Jul 26;11:234. doi: 10.1038/s41377-022-00924-3 (PMC9325779; doi:10.1038/s41377-022-00924-3)
Supplement: Supplementary file 1 — Supplementary Information [file 41377_2022_924_MOESM1_ESM.pdf]

Supplementary Information for
Data-driven Design of High-performance $MASn_xPb_{1-x}I_3$ Perovskite Materials by Machine Learning and Experimental Realization

Xia Cai,[1,2,3] Fengcai Liu,[1,3] Anran Yu,[1,3] Jiajun Qin,[4] Mohammad Hatamvand,[1,3] Irfan Ahmed,[1,3] Jiayan Luo,[1,3] Yiming Zhang,[1,5] Hao Zhang,[1,5,6,*] and Yiqiang Zhan[1,3,†]

[1]*School of Information Science and Technology, Fudan University, Shanghai 200433, China*

[2]*College of Information, Mechanical and Electrical Engineering, Shanghai Normal University, Shanghai, 200234, China*

[3]*Center of Micro-Nano System, Fudan University, Shanghai 200433, China*

[4]*Department of Physics, Chemistry and Biology, Linköping University, Linköping SE-58183, Sweden*

[5]*Key Laboratory of Micro and Nano Photonic Structures and Department of Optical Science and Engineering, Fudan University, Shanghai 200433, China*

[6]*Yiwu Research Institute of Fudan University, Chengbei Road, Yiwu City, Zhejiang 322000, China*

SUPPLEMENTARY NOTES

Model description: Linear regression (LR) is the simplest model in ML, which fits a linear model to minimize the residual sum of squares between the observed responses in the dataset. K-nearest neighbor regression (KNR) is based on k nearest neighbors (k is the number to be set by the user) to represent the data for learning. The primary hyper-parameters of KNR are the number of neighbors, weight function used in prediction and algorithm used to compute the nearest neighbors. k is selected from 1,2,...,30, weight function is uniform, and algorithm is selected from 'auto', 'ball_tree', 'kd_tree' and 'brute'. Support vector regression (SVR) can consider the balance between empirical risk and expected risk to achieve good prediction and generalization. Through introducing the kernel function, it can convert the original data into the required feature space to solve the complicated nonlinear regression problems. The primary hyper-parameters of SVR are penalty parameter C of the error term, kernel type and kernel coefficient. C is uniformly selected from $[0, 3]$, kernel type is selected from 'linear' and 'rbf', and kernel coefficient is uniformly generated from $[0, 20]$. Random forest regression (RFR) based on decision tree algorithm is an ensemble learning model that can effectively avoid over-fitting, but cannot predict data outside the training data range. Gradient boosting regression (GBR) is also one of the tree-based ML algorithms, which is based on the gradient boosting method and is a single strong learner through combining many weak learners step by step using the gradient descent algorithm. It has strong prediction ability and is robust to outliers in output space. RFR and GBR are both based on tree arithmetic, the primary hyper-parameters are the number of trees (selected from 1,2,...,100), the maximum depth of the tree (selected from 1,2,...,20), the minimum number of samples required to split an internal node (selected from 2,3,...,20) and the minimum number of samples required to be at a leaf node (selected from 1,2,...,20). GBR has another hyper-parameter, learning rate, which is uniformly generated from $[1e-3, 9e-1]$. The implementation of above hyper-parameter selection is based on a Python library called hyperopt. In recent years, neural networks (NN) have been widely used in deep learning with strong learning capability of non-linear models. In our NN model, learning rate is set to 0.003, neuron type is tanh function and two hidden layers are used with 64 and 32 neurons, respectively. Bayesian Optimization algorithm based on Bayes Theorem leads a search of a global optimization problem. It works by building a probabilistic model of the objective function, which is then searched efficiently with an acquisition function before candidate samples are chosen for evaluation on the real objective function. Genetic algorithm is a search

heuristic where through the process of selection, crossover and mutation a population of candidate solutions (called individuals) to an optimization problem is evolved toward better solutions based on the results of fitness function.

Performance evaluation: The coefficient of determination (R^2), root mean square error (RMSE) and mean absolute error (MAE) were used as the evaluation metrics of models. And the model with higher R^2 and smaller RMSE and MAE is better.

$$R^2 = 1 - \frac{\sum_{i=1}^{N}(y_i^{true} - y_i^{pred})^2}{\sum_{i=1}^{N}(y_i^{true} - \bar{y}_i^{pred})^2} \quad (1)$$

$$RMSE = \sqrt{\frac{\sum_{i=1}^{N}(y_i^{true} - y_i^{pred})^2}{N}} \quad (2)$$

$$MAE = \frac{1}{N}\sum_{i=1}^{N}\left|y_i^{true} - y_i^{pred}\right| \quad (3)$$

N is the number of samples, y_i^{true} is the actual value, y_i^{pred} is the predicted value and $\bar{y}_i^{pred}$ is the mean value of y^{pred}.

SUPPLEMENTARY TABLES AND FIGURES

TABLE S1. The 14 features in band gap regression model of perovskite material.

No.	Symbol	Property
1	R^{ion}	Ionic radii of B-site
2	E^{ip}	First ionization energy of B-site
3	E^{ea}	Electron affinity of B-site
4	E^{en}	Mulliken's electronegativity of B-site
5	N^{men}	Mendeleev's number of B-site
6	$HOMO$	HOMO for B-site
7	$LUMO$	LUMO for B-site
8	P	Polarizability of B-site
9	ρ	Density of B-site metal
10	T_f	Tolerance factor defined as $\frac{R_A+R_X}{\sqrt{2}(R_{\bar{B}}+R_X)}$
11	α_o^3	Unit cell lattice edge
12	R^c	Critical radii
13	R_a^{ion}/R_b^{ion}	Ratio between the ionic radii of A and B site
14	M	The weight of perovskite

TABLE S2. The prediction performance of different regression algorithms for four targets (FF, J_{sc}, V_{oc} and PCE) in designing PSCs device using predicted values of E_g. The best results are highlighted in **bold** and NN almost behaves the best.

Method	FF			J_{sc}			V_{oc}			PCE		
	R^2	RMSE	MAE	R^2	RMSE	MAE	R^2	RMSE	MAE	R^2	RMSE	MAE
LR	0.4173	8.6426	5.8037	0.0231	5.0775	2.8394	0.6996	0.1173	0.0799	0.4735	3.5899	2.4003
SVR	0.5150	**7.8847**	**4.2509**	0.1492	4.7385	2.5488	0.7105	0.1151	0.0873	0.5639	3.2674	2.1164
KNR	0.4721	8.2265	5.3905	0.1063	4.8565	2.8878	0.7370	0.1097	0.0745	0.5628	3.2714	2.1804
RFR	0.4924	8.0666	5.8874	-0.1483	5.5049	3.4574	0.6638	0.1240	0.0784	0.3572	3.9669	2.6161
GBR	0.4865	8.1136	5.6405	0.1422	4.7580	2.8714	0.8175	0.0914	0.0667	0.6169	3.0625	2.2012
NN	**0.7251**	7.9573	5.8238	**0.8877**	**2.1994**	**1.4251**	**0.8909**	**0.0892**	**0.0628**	**0.8889**	**2.0964**	**1.6705**

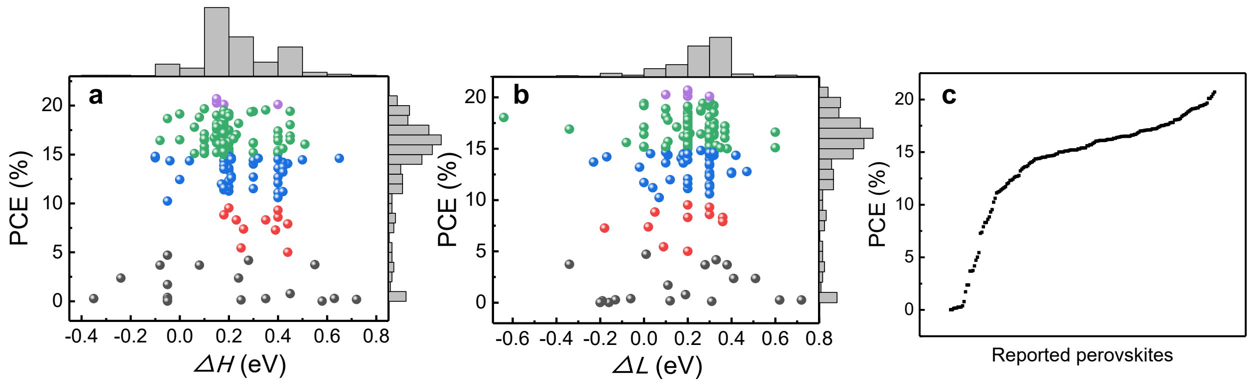

FIG. S1. The marginal histograms of a) PCE versus energy difference (ΔH); b) PCE versus energy difference (ΔL). Different colors represent different regions of PCE values. c) PCE distribution of 181 collected perovskites in the performance model. For data collection, the terms of the device fabrication are refined as follows. All materials and precursors received are without further purification from companies including Sigma-Aldrich, TCI, Heraeus Clevios, Greatcell Solar, etc., to meet the quality standard. Self-synthesized materials are only taken into account after the synthetic approach was reported at least in two independent works. The $MASn_xPb_{1-x}I_3$ perovskite films are deposited in glove box filled with inert gas where O_2 and H_2O both are less than 0.01 ppm and atmosphere temperature is at 25°C±5°C. The annealing temperature of as-deposited films is locked within 100°C to 125°C. For solution process, deposition approach is only in anti-solvent and sequential method with DMF, DMSO, NMP and GBL as four main solvents applied independently or in combine. For the experimental verification part, we also adapt all these standards into device fabrication.

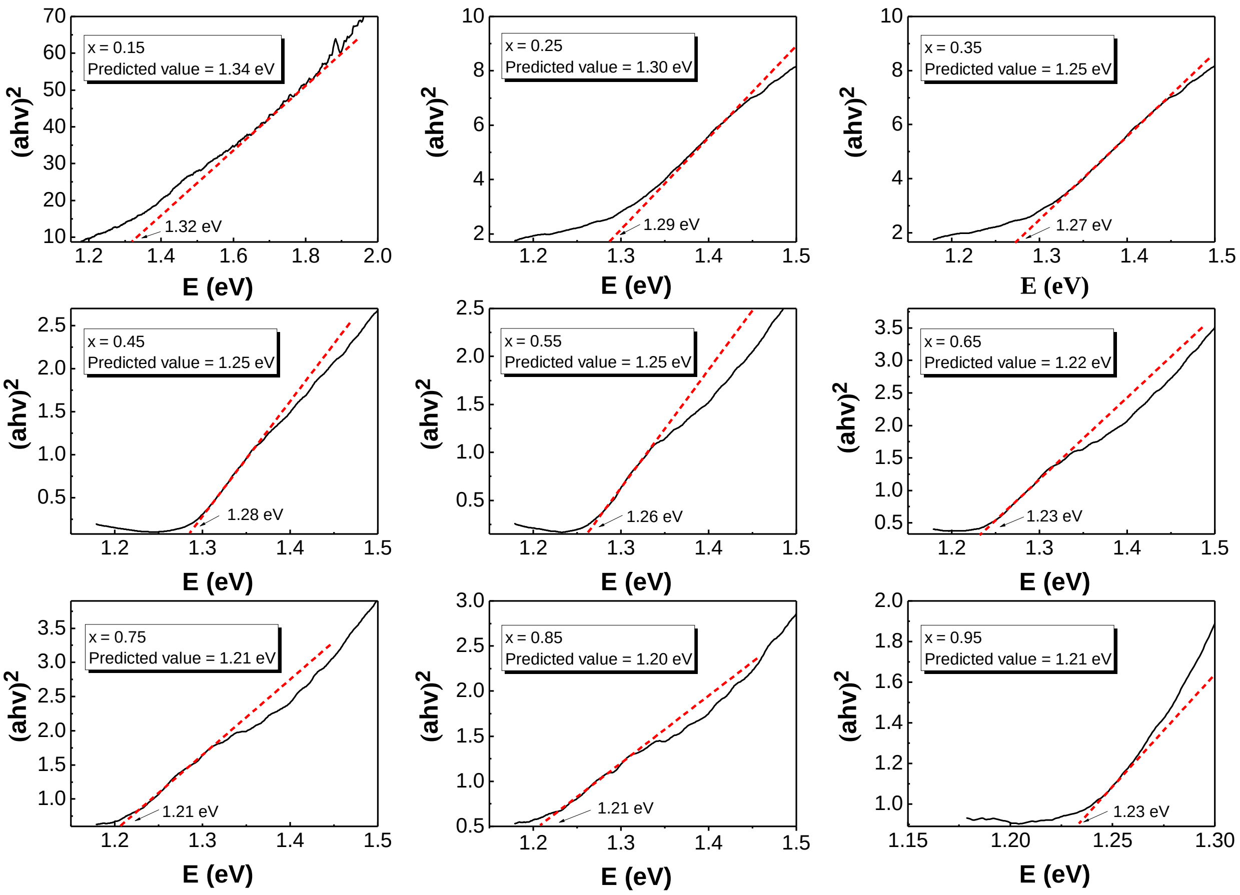

FIG. S2. Absorption spectra of fabricated $MASn_xPb_{1-x}I_3$ with different fraction of x according to ML predictions.

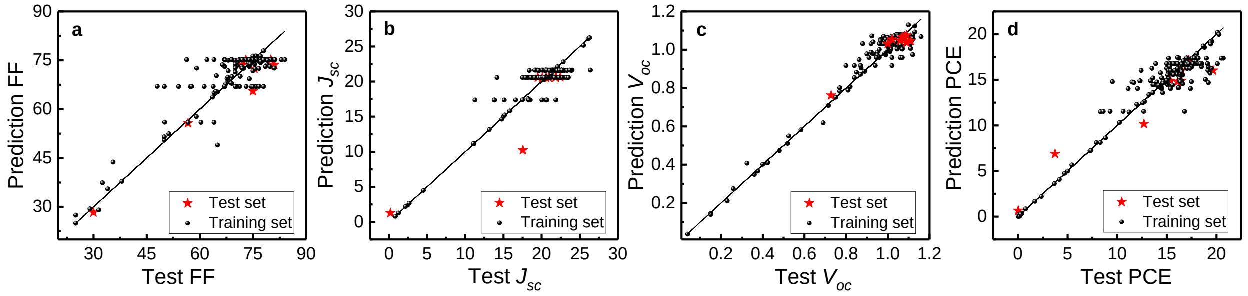

FIG. S3. The prediction performances of PSC design models by NN using actual E_g values. Training set and test set are plotted with actual values and predicted values in black dots and red stars, respectively. a) Predicted FF versus actual FF. b) Predicted J_{sc} versus actual J_{sc}. c) Predicted V_{oc} versus actual V_{oc}. d) Predicted PCE versus actual PCE. The black line indicates the predictions are equal actual values. The closer the dots to the black line means the model has higher accuracy.

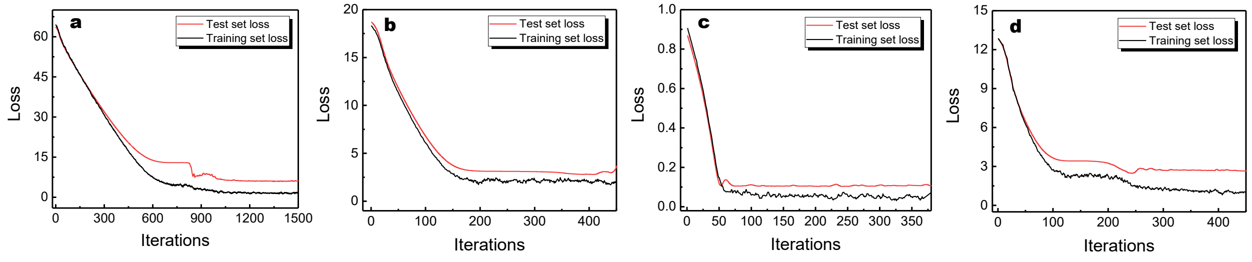

FIG. S4. The loss of training and test set of PSC design models during NN training plotted in black and red lines, respectively, for a) FF model; b) J_{sc} model; c) V_{oc} model; d) PCE model.

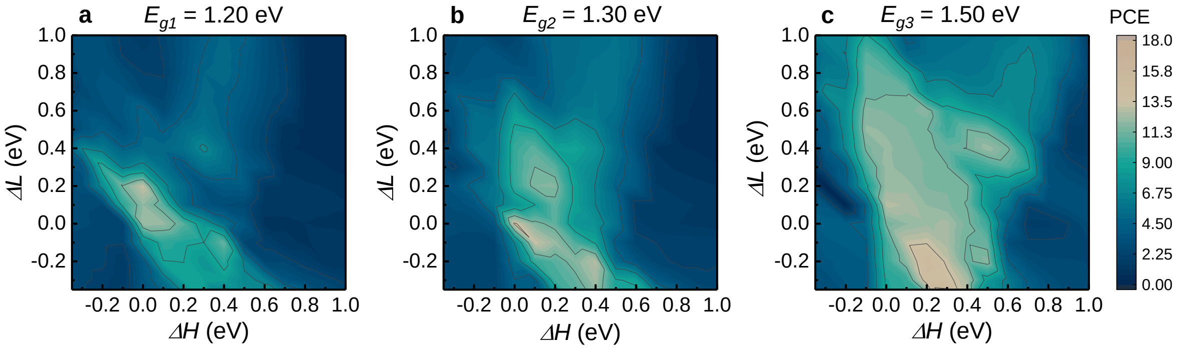

FIG. S5. 2D-contour map of calculated PCE at a) E_{g1}= 1.20 eV; b) E_{g2}= 1.30 eV; c) E_{g3} = 1.50 eV.

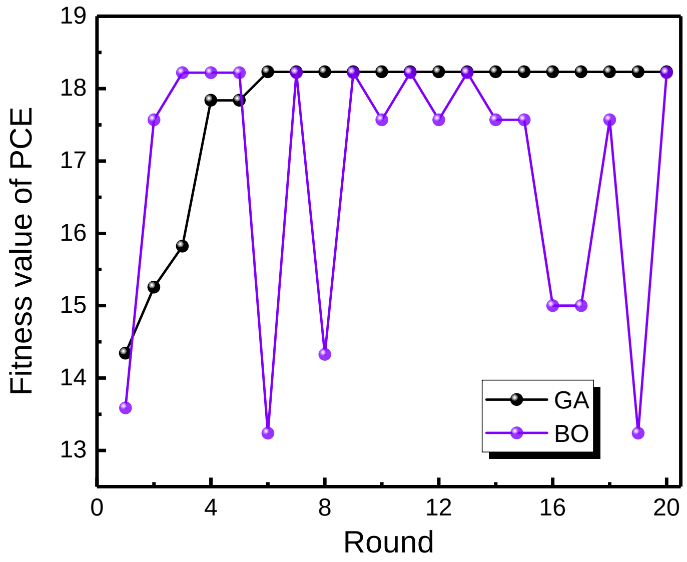

FIG. S6. The fitness value of PCE against the search round by GA and BO explorations.

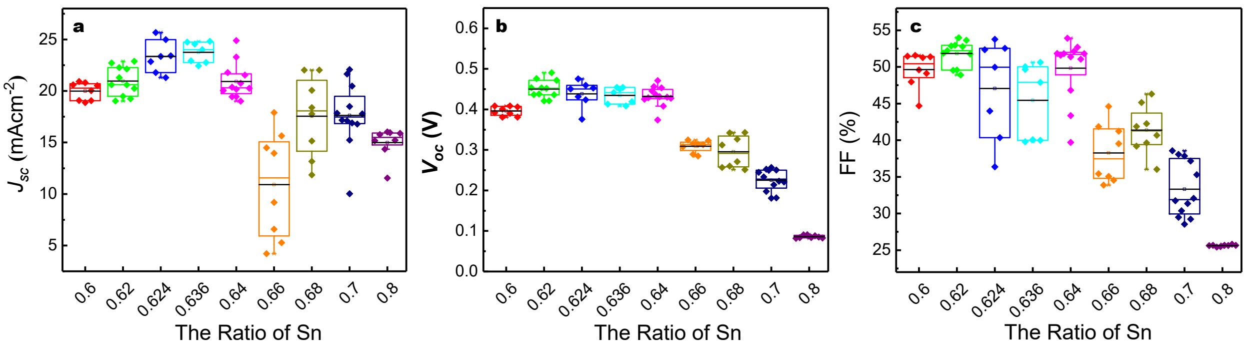

FIG. S7. The experimental (a) J_{sc}, (b) V_{oc} and (c) FF of $MASn_xPb_{1-x}I_3$ solar cells with different Sn contents.

TABLE S3. Summary of the content of Sn greater than 0.5 in mixed Sn-Pb single-junction perovskite solar cells, which are all prepared by one-step method.

The ratio of Sn	PCE	Regular	Inverted	Reference
0.7	0.13%	✓		[1]
0.75	3.74%	✓		[2]
1.0	5.44%	✓		[2]
0.75	2.36%		✓	[3]
1.0	0.26%		✓	[3]
**0.6**	**10.0%**		✓	[4]
0.8	7.6%		✓	[4]
1.0	3.2%		✓	[4]
1.0	2.56%		✓	[5]
1.0	6.63%	✓		[6]
1.0	0.11%	✓		[7]

* zhangh@fudan.edu.cn

† yqzhan@fudan.edu.cn

[1] Y. Ogomi, A. Morita, S. Tsukamoto, T. Saitho, N. Fujikawa, Q. Shen, T. Toyoda, K. Yoshino, S. S. Pandey, T. Ma *et al.*, *The journal of physical chemistry letters*, 2014, **5**, 1004–1011.

[2] F. Hao, C. C. Stoumpos, R. P. Chang and M. G. Kanatzidis, *Journal of the American Chemical Society*, 2014, **136**, 8094–8099.

[3] Z. Yang, A. Rajagopal, C.-C. Chueh, S. B. Jo, B. Liu, T. Zhao and A. K.-Y. Jen, *Advanced Materials*, 2016, **28**, 8990–8997.

[4] B. Zhao, M. Abdi-Jalebi, M. Tabachnyk, H. Glass, V. S. Kamboj, W. Nie, A. J. Pearson, Y. Puttisong, K. C. Gödel, H. E. Beere *et al.*, *Advanced materials*, 2017, **29**, 1604744.

[5] J. Zhang, T. Wu, J. Duan, M. Ahmadi, F. Jiang, Y. Zhou and B. Hu, *Nano energy*, 2017, **38**, 297–303.

[6] W. Ke, C. C. Stoumpos, I. Spanopoulos, L. Mao, M. Chen, M. R. Wasielewski and M. G. Kanatzidis, *Journal of the American Chemical Society*, 2017, **139**, 14800–14806.

[7] F. Li, C. Zhang, J.-H. Huang, H. Fan, H. Wang, P. Wang, C. Zhan, C.-M. Liu, X. Li, L.-M. Yang *et al.*, *Angewandte Chemie International Edition*, 2019, **58**, 6688–6692.
